# Supplementary material for: Corrosion Resistance of Aluminum against Acid Activation: Impact of Benzothiazole-Substituted Gallium Phthalocyanine
Source: Molecules. 2019 Jan 8;24(1):207. doi: 10.3390/molecules24010207 (PMC6337598; doi:10.3390/molecules24010207)
Supplement: Supplementary file 1 [file molecules-24-00207-s001.pdf]

Supporting Information

Corrosion resistance of aluminium against acid activation: Impact of benzothiazole substituted gallium phthalocyanine

Nnaemeka Nnaji, Njemuwa Nwaji, John Mack and Tebello Nyokong*

Centre for Nanotechnology Innovation, Department of Chemistry, Rhodes University, Grahamstown 6140, South Africa

Inhibition efficiencies:

The inhibition efficiencies (IE%) were calculated using equation reported before [1,2]

$$IE\% = \frac{I^0_{\mathrm{corr}} - I^{\mathrm{i}}_{\mathrm{corr}}}{I^0_{\mathrm{corr}}} \times 100 \qquad \text{S1}$$

where I^0_{corr} and I^i_{corr} (determined form Fig. S1) are the corrosion current density values in the absence and in the presence of inhibitor, respectively.

Adsorption isotherm equations

Below are expressions [3,4] for the adsorption isotherms used to fit the adsorption data of BTThio and ClGaBTThioPc onto aluminium in hydrochloric acid such that the result obtained helped to describe the nature of adsorption.

$$C / \theta = C / K_{\mathrm{L}} + C \qquad \text{(Langmuir)} \qquad \text{S2}$$

$$ln\,\theta = ln\,K_{\mathrm{F}} + 1 / n\,ln\,C \qquad \text{(Feundlich)} \qquad \text{S3}$$

$$\theta = 1/f(ln K_T + ln C) \qquad \text{(Temkin)} \qquad S4$$

$$ln[(\frac{\theta}{1-\theta})] = ln K_{El} + Y_{El}\, ln C) \qquad \text{(El-Awady)} \qquad S5$$

$$ln[C(\frac{\theta}{1-\theta})] = ln K_{Fr} + 2\alpha\theta \qquad \text{(Frumkin)} \qquad S6$$

where equations S2-S5 represent Langmuir, Freundlich, Temkin, El-Awady and Frumkin adsorption isotherms, respectively [3,4]. Symbols presented by the adsorption isotherm equations are: the degree of surface coverage (θ), equilibrium adsortion constants or adsorption capacity values (K_L, K_F, K_{Fr}, K_T and K_{El}) and adsorption parameters (n and Y_{El}) which aid to characterise the nature of inhibitor adsorption.

The determination of standard free energy of adsorption (ΔG^0_{ads}), however, was obtained using equation S6 [5]:

$$K_{ads} = \frac{e^{-\frac{\Delta G^0_{ads}}{RT}}}{55.5} \qquad S7$$

where R is the gas constant and T is the temperature in K. The standard free energy of adsorption, ΔG^0_{ads}, characterizes the interaction of adsorption molecules on metal surface and was calculated using equation S6 from the equilibrium adsorption contants (K_L, K_F, K_T and K_{El}) of the isotherm plots. Negative values of ΔG^0_{ads} calculated aid to decide the spontaneity of adsorption process and stability of the adsorbed layer on the

metal surface. Generally, the values of ΔG^0_{ads} around −20 kJ/mol or less in magnitude are consistent with physisorption and those around −40 kJ/mol or higher in magnitude involve chemisorptions [6].

The inhibition efficiency (IE%) values from impedance measurements were calculated using equation reported before [7]

$$IE\% = \frac{R_{t,i} - R_{t,0}}{R_{t,i}} \times 100 \qquad \text{S8}$$

where $R_{t,i}$ and $R_{t,0}$ are the charge transfer resistances in the presence and absence of the inhibitors, respectively.

Quantum chemical studies

Electronegativity (χ) is the meaasure of the power of an electron or group of atoms to attract electrons

towards itself and it can be estimated by using the equation S9:

$$\chi \cong -½\ (E_{HOMO} + E_{LUMO}) \qquad \text{S9}$$

Global hardness (η) measures the resistance of an atom to a charge transfer and was estimated using equation S10:

$$\eta \cong -½\ (E_{HOMO} - E_{LUMO}) \qquad \text{S10}$$

Global softness (σ), describes the capacity of an atom or group of atoms to receive electrons [8], it was estimated by using the equation S11:

$$\sigma = 1/\eta \cong -2/(E_{HOMO} - E_{LUMO}) \qquad \text{S11}$$

$$\Delta N = \frac{\chi_{Al} - \chi_{inh}}{2(\eta_{Al} + \eta_{inh})} \qquad \text{S11}$$

where $\chi_{Al} \approx$ 3.23 eV and $\eta_{Al} = 0$ [9].

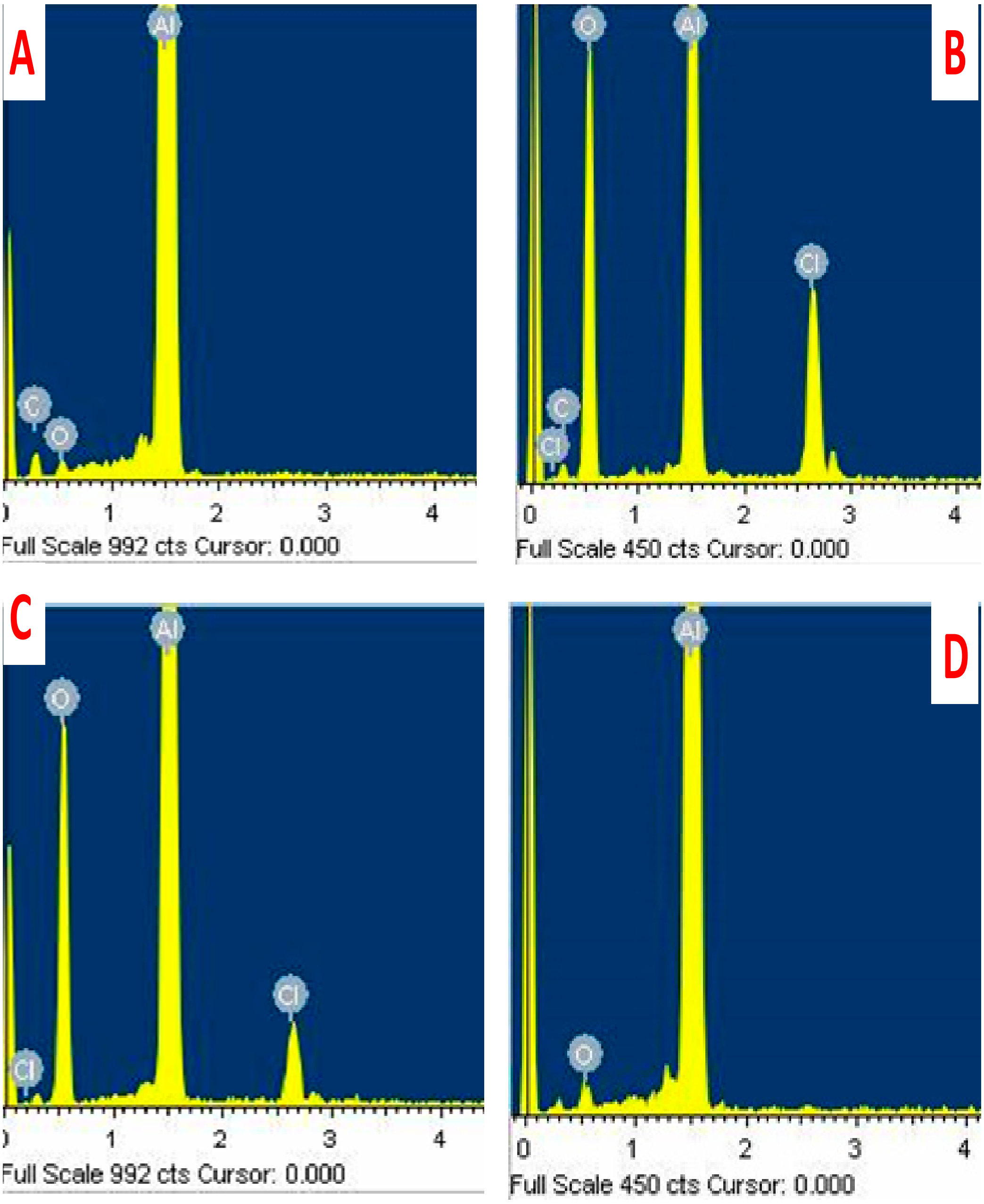

Figure S1: EDX of (A) bare aluminium metal, (B) aluminium metal after immersion in 1.0 M HCl, (C) aluminium metal after immersion in 1.0 M HCl containing 6.0 μM BTThio, and (D) aluminium metal after immersion in 1.0 M HCl containing 6.0 μM ClGaBTThioPc.

References

[1] Mishra, A.K.; Balasubramanian, R. Corrosion inhibition of aluminum alloy AA 2014 by rare earth chlorides. Corr. Sci. 2007, 49, 1027-1044, doi.org/10.1016/j.corsci.2006.06.026..

[2] Nnaji, N.J.N.; Ujam, O.T.; Ibisi, N.E.; Ani, J.U.; Onuegbu, T.O; Olasunkanmi, L.O.; Ebenso, E.E. Morpholine and piperazine based carboxamide derivatives as corrosion inhibitors of mild steel in HCl medium. *J. Mol. Liq.* 2017, 230, 652-661, doi.org/10.1016/j.molliq.2017.01.075.

[3] Abd El-Rehim, S.S.; Refaey, S.A.M.; Taha, F.; Saleh, M.B.; Ahmed, R.A. Corrosion Inhibition of Mild Steel in Acidic Medium using 2-amino Thiophenoland 2-Cyanomethyl Benzothiazole. *J. Appl. Electrochem.* 2001, 31, 429-435, DOI not available.

[4] Abd El Rehim, S.S.; Sayyah, S.M.; El-Deeb, M.M.; Kamal, S.M.; Azooz, R.E. Adsorption and corrosion inhibitive properties of P(2-aminobenzothiazole) on mild steel in hydrochloric acid media. Int. J. Ind. Chem. 2016, 7, 39–52, DOI 10.1007/s40090-015-0065-5.

[5] Shukla, S.K.; Ebenso, E.E. Corrosion Inhibition, Adsorption Behavior and Thermodynamic Properties of Streptomycin on Mild Steel in Hydrochloric Acid Medium. Int. J. Electrochem. Sci. 2011, 6, 3277-3291, DOI not available.

[6] Salarvand, Z.; Amirnasr, M.; Talebian, M; Raeissi, K.; Meghdadia, S. Enhanced corrosion resistance of mild steel in 1 M HCl solution by trace amount of 2-phenyl-benzothiazole derivatives: Experimental, quantum chemical calculations and molecular dynamics (MD) simulation studies. *Corros. Sci*. 2017, 114, 133-145, doi.org/10.1016/j.corsci.2016.11.002.

[7] Yang, D.; Zhang, M.; Zhenga, J.; Castaneda, H. Corrosion inhibition of mild steel by an imidazolium ionic liquid compound: the effect of pH and surface pre-corrosion. RSC Adv., 2015, 5, 95160–95170, DOI: 10.1039/c5ra14556b.

[8] Ögretir, C.; Demirayak, S.; Tay, N.F.; Duran, M. Determination and Evaluation of Acid Dissociation Constants of Some Substituted 2-Aminobenzothiazole Derivatives. J. Chem. Eng. Data. 2008, 53, 422-426, DOI: 10.1021/je700515k.

[9] John, S.; Joseph, A. Quantum chemical and electrochemical studies on the corrosion inhibition of aluminium in 1 N HNO_3 using 1,2,4-triazine. Materials and Corros. 2013, 64, 625-632, DOI: 10.1002/maco.201206782.
